# “Like I’m Talking to a Real Person”: Exploring the Meaning of Transference for the Use and Design of AI-Based Applications in Psychotherapy

**DOI:** 10.3389/fpsyg.2021.720476

**Published:** 2021-09-27

**Authors:** Michael Holohan, Amelia Fiske

**Affiliations:** Institute of History and Ethics in Medicine, School of Medicine, Technical University of Munich, Munich, Germany

**Keywords:** artificial intelligence, psychotherapy, mental healthcare, chatbots, transference, embedded ethics, science and technology studies, agential realism

## Abstract

AI-enabled virtual and robot therapy is increasingly being integrated into psychotherapeutic practice, supporting a host of emotional, cognitive, and social processes in the therapeutic encounter. Given the speed of research and development trajectories of AI-enabled applications in psychotherapy and the practice of mental healthcare, it is likely that therapeutic chatbots, avatars, and socially assistive devices will soon translate into clinical applications much more broadly. While AI applications offer many potential opportunities for psychotherapy, they also raise important ethical, social, and clinical questions that have not yet been adequately considered for clinical practice. In this article, we begin to address one of these considerations: the role of transference in the psychotherapeutic relationship. Drawing on Karen Barad’s conceptual approach to theorizing human–non-human relations, we show that the concept of transference is necessarily reconfigured within AI-human psychotherapeutic encounters. This has implications for understanding how AI-driven technologies introduce changes in the field of traditional psychotherapy and other forms of mental healthcare and how this may change clinical psychotherapeutic practice and AI development alike. As more AI-enabled apps and platforms for psychotherapy are developed, it becomes necessary to re-think AI-human interaction as more nuanced and richer than a simple exchange of information between human and nonhuman actors alone.

## Introduction

A first-year college student is having trouble adjusting to university life. There are so many new things to deal with, so many new demands and responsibilities. She is making new friends, but finds it hard to connect with them. Her grades are starting to slip and she feels like she is losing control of her life. When she eventually decides to check the campus health service website to see what mental health services are available, she finds that there is a long waitlist to see a counselor. However, the website suggests an alternative that is available immediately and is entirely free: a text-based chatbot, powered by artificial intelligence (AI). Using an app the student downloaded onto her phone, the chatbot checks in regularly to ask how she is doing, helps her to identify the emotions she feels in difficult situations, and suggests some relaxation exercises to work through her anxiety. She likes that the chatbot is available around the clock and always texts back immediately. Even though she knows she is talking to a computer, she feels heard and even understood.

Like this example, chatbots such as Tess,^1^ Wysa,^2^ or Woebot^3^ offer similar virtual psychotherapeutic services and have demonstrated promising results in reducing symptoms of depression and anxiety in trial studies (Fitzpatrick et al., 2017; Fulmer et al., 2018). AI-enabled virtual and robot therapy is increasingly being integrated into psychotherapeutic practice. Given the speed of research and development trajectories of AI-enabled applications in psychotherapy and the practice of mental healthcare, it is likely that therapeutic chatbots, avatars, and socially assistive devices will soon translate into clinical applications much more broadly.

However, this field is still nascent and there are many questions that remain to be considered or clarified. For example, what does it mean to interact with a robot for help with your mental health? What does it mean to form a personal connection in a therapeutic setting with something you know is not a person? This is an issue not just for the users who access these services, but also for the engineers and designers who are developing these interfaces: How to best design algorithms that help people work through their intimate problems in a way that fosters a connection between the person and the interface? How is the therapeutic connection established and how do you factor it into your design? Moreover, is the nature of the connection with a virtual therapist even comparable to that of a human therapist?

The companies developing AI-enabled therapeutic applications have designed the applications to look and feel much like in-person therapy. However, this surface similarity obscures the possibility that there may be significant differences between AI-directed and human-directed psychotherapy. Therefore, it is necessary to carefully examine the points of similarity and difference between AI-directed and human-directed psychotherapy. Doing so will allow us to better understand not only the limitations of AI applications vis-a-vis traditional psychotherapy, but also what is new and unique about such applications and what they might make possible.

One aspect that deserves particular attention is the sense of “personal” connection between user-patients and their chatbot therapist. This is because most modalities of psychotherapy have a concept of “transference,” which describes a specific way that patients and therapists relate to each other within the therapeutic relation. In this article, we focus on transference as one example in order to highlight some fundamental issues related to the use of AI-enabled psychotherapy. Drawing on the work of Science and Technology Studies (STS)^4^ scholar Karen Barad on material-discursive practices in human–non-human relations (Barad, 1999, 2007), we present a framework for conceptualizing the therapeutic setting in order to help those involved (psychotherapists, patients, support staff, caretakers, robotics engineers, developers, researchers, ethicists, administrators, legislators, etc.) better understand the nature of the AI-driven therapeutic encounter. This approach can help to inform further work in this field, in terms of therapeutic practice with existing AI applications, research into the effects of such practices, and the research and development of new AI applications.

In what follows, we first present a review of the literature on existing AI-enabled psychotherapeutic applications. We then outline the concept of transference in psychotherapy, putting it in conversation with Barad’s theory of agential realism. We end with a discussion of the implications of transference in relation to AI-enabled psychotherapy and possibilities for further research.

## The Current State of AI-Enabled Psychotherapeutic Applications

Work in embodied artificial intelligence (AI) has growing clinical relevance for diagnostic and therapeutic applications across several areas in medicine (Calderita et al., 2014; Broadbent, 2017; Liu et al., 2018). Such applications are no longer designed to just provide simple assistive services, but also perform higher-level, invasive, diagnostic, and therapeutic interventions that used to be offered exclusively by highly trained health professionals (Jahn et al., 2019). In the area of mental health, embodied AI is increasingly being integrated into psychotherapeutic practice (Fiske et al., 2019). It has been proposed to support a range of emotional, cognitive, and social processes (Eichenberg and Küsel, 2018) through the use of chatbots, virtual reality therapies, social robots, and more. In what follows, we briefly summarize the range of AI applications that are being researched, tested, and applied in the area of mental healthcare, with a specific focus on applications within psychotherapy. As such, we have intentionally excluded from this analysis all AI applications that do not interact with patients directly, and those that may have a virtual or robotic interface but do not employ AI, such as telemedicine therapy.

The most prominent domain of AI-driven psychotherapeutic applications is therapeutic apps, sometimes called “chatbots.” Known by their first names, apps such as Tess, Sara, Wysa, Ada, or Woebot work *via* text or on internet platforms and have addressed conditions such as depression, anxiety, and autism. Many such applications respond to the user in a way that aims to mimic a human therapist, probing the user to explore emotions or thought patterns that they are experiencing. Others offer techniques for reducing anxiety or advice for dealing with difficult situations (Sachan, 2018; Dekker et al., 2020), help users implement problem-solving strategies and approach problems from different perspectives, or inform users of nearby psychiatric services when needed (Bendig et al., 2019). Recent reviews found over 40 chatbots addressing mental health concerns available, most with several purposes including therapy, training, and screening (Abd-alrazaq et al., 2019; Tudor Car et al., 2020).

The area of virtual reality is increasingly being proposed for use with patients experiencing psychosis (Craig et al., 2018), schizophrenia, and autism. One such example currently in clinical testing is the Avatar Project,^5^ in which an intelligent algorithm is expressed through an avatar which interacts with a patient in order to address symptoms such as persistent auditory hallucinations. The use of avatars is also being explored in AI-assisted therapy for schizophrenia (Dellazizzo et al., 2018a,b) as well as in combination with real-time fMRI (de Pierrefeu et al., 2018). Studies of virtual human agents have also experimented with improving interviewing skills with individuals with autism or other developmental disabilities (Burke et al., 2018), promoting life skills and well-being for adolescents (Gabrielli et al., 2020), treating the fear of heights (Freeman et al., 2018; Donker et al., 2019), and risk prevention (Rein et al., 2018).

While some technologies might be used as part of supervised therapies, AI-driven psychotherapeutic applications such as chatbots are slowly but surely progressing toward a therapeutic role outside of settings where human mental health professionals are involved. It is therefore necessary to assess how important elements of the “traditional” relationship between therapist and client/patient are either retained, altered, or made anew in the relationship between user and chatbot therapist. One central element of the traditional therapeutic relationship is transference, which is of particular interest because it is a form of personal connection that is specific to the psychotherapeutic setting. In the next section, we will discuss the concept of transference, how it functions, and why it is relevant for the study and design of AI-directed therapies.

## The Concept of Transference in Psychotherapy

The concept of transference can be traced back to the earliest days of psychotherapy. Introduced by Sigmund Freud (1912/2001) in the context of psychoanalytic treatment, it is a foundational concept in many forms of psychotherapy. Transference refers to a phenomenon where a patient redirects emotions, feelings, or wishes that were originally directed toward other people in their life onto the therapist (Goldstein and Goldberg, 2004; Parth et al., 2017). Transference can manifest, for example, in a patient’s speech, demeanor, attitude, or patterns of behavior (Fink, 2007). The appearance of transference is not an accident, but an inevitable aspect of the therapeutic process (Freud, 1912/2001; Friedman, 2019). Put another way: it is not a bug; it is a feature of the therapeutic relationship.

Transference is integral to the interpersonal relationship between patient and therapist and represents an important point of action in the psychotherapeutic process. Regardless of what the two parties are talking about at a given moment, there is always another relationship in the room, i.e., the patient’s relationship to someone else in their life, either actual or imagined. However the patient speaks to and acts toward their therapist—including silences and elisions—the past is present in their speech and behavior in the form of these prior relationships that the patient brings (i.e., transfers) into the consulting room. The therapist must be able to acknowledge this transference and work with it, since it is as indispensable to the treatment as it is unavoidable. Transference can have multiple different effects in the therapeutic relationship. For example, transference can help foster the therapeutic alliance, especially in the early stages of the treatment. A positive transference can make it possible for the patient to face difficult subjects, by helping them feel supported and understood. Transference is often also an object of analysis itself, and identifying, discussing, and actively working through transference feelings is a significant part of most psychodynamic psychotherapies. Transference can also act as a form of resistance and as an obstacle to treatment by keeping the patient from feeling like they can discuss certain ideas or topics, or a strong negative transference can make it hard for the patient to attend sessions regularly or even cause them to terminate the treatment (for an overview of the different effects of transference and ways of working with it, see Fink, 1997; Corradi, 2006; and Fink, 2007, esp. chapter 7; for empirical studies of its usefulness, see Marmarosh, 2012; Hersoug et al., 2014; Suszek et al., 2015; Ulberg et al., 2021).

Depending on the specific theoretical orientation of the psychotherapy, working with transference may be more or less central to the treatment, but it nonetheless remains a tool in the therapist’s tool kit. For example, imagine a patient for whom the therapist’s haircut or tone of voice resembles the hair or voice of her father, with whom she has a poor relationship. Based on this trivial similarity, the patient begins, sometimes without even meaning to, to act toward her therapist with the same kind of denial and protest that she did with her father. This transference of feeling from the father onto the therapist can lead the patient to complain about the therapist, find it hard to trust him, or even start to miss sessions. Without identifying and working through this negative transference, the therapy is unlikely to make any progress. It is worth noting that while we have referred here to “positive” or “negative” transference feelings, more often transference represents a fusion of contradictory currents (positive and negative, love and hate, admiration and fear, etc.) that are inextricably entangled with each other.

While the concept of transference is most commonly associated with psychoanalytic and psychodynamic psychotherapies, it is also discussed in other approaches such as cognitive behavioral therapy (CBT) (Prasko et al., 2010; Folk et al., 2016). This is particularly significant because existing chatbots like Woebot and Tess are designed on CBT principles (Fitzpatrick et al., 2017; Fulmer et al., 2018).

As of yet, there have been no studies of transference in AI-enabled psychotherapeutic settings. However, studies of specific chatbots demonstrate anecdotal evidence that some users develop a human-like connection with the chatbot that can be seen as suggestive of the kind of personal relationship out of which transference can develop. For example, one study participant wrote, “I love Woebot so much. I hope we can be friends forever. I actually feel super good and happy when I see that it ‘remembered’ to check in with me!” (Fitzpatrick et al., 2017). Another participant in a similar study of the chatbot Tess wrote “Based on our interactions I do somewhat feel like I’m talking to a real person and I do enjoy the tips you’ve given. In that sense, you’re better than my therapist in that she doesn’t necessarily provide specific ways I can better myself and problems” (Fulmer et al., 2018).

## Understanding Psychotherapy Through the Lens of Karen Barad’s Agential Realism

Transference is both a product of the psychotherapeutic encounter and a mechanism though which treatment occurs. In order to better understand what this means and how it can be considered in AI-driven therapy, we turn to STS scholar Karen Barad’s theory of agential realism, a conceptual approach to theorizing human–non-human relations (Barad, 1999, 2007). Barad’s theory provides a framework for conceptualizing and understanding what the psychotherapeutic encounter consists of, what its elements are, and how those elements shape what is possible in the encounter. This makes it possible to analyze different kinds of situations and identify how substituting a chatbot for a human therapist might alter the situation. Barad’s theory focuses on knowledge production, which relates to AI-driven psychotherapy in terms of how it creates knowledge about such things as emotional states, patterns of behavior, or unconscious desires, depending on the therapeutic tradition.

Barad builds on the theoretical and epistemological work of quantum physicist Niels Bohr, arguing that the knower does not stand apart from the object they seek to measure (Barad, 2007). As an illustrative example, she considers the well-known Heisenberg uncertainty principle, which states that it is impossible to measure both a particle’s position and velocity at the same time. Bohr argued that this is because the experimental apparatus determines what can be measured and thus also the conceptual framework for understanding.

For example, instruments with fixed parts are required to understand what we might mean by the concept ‘position.’ However, any such apparatus necessarily excludes other concepts, such as ‘momentum,’ from having meaning during this set of measurements, since these other variables require an instrument with moveable parts for their definition. Physical and conceptual constraints are co-constitutive. (Barad, 1999, p. 4)

The interaction between what is observed and the apparatus used to observe it are thus inseparable from each other. Together they produce what Barad calls phenomena, and these phenomena are constitutive of the apparatus as well as the products of that apparatus, by means of “physical-conceptual *intra-actions*” (Barad, 1999, p. 5).

An apparatus is the set of materials and practices that, by being put to use in a specific situation and for a specific purpose, create the conditions of possibility for what can happen in that situation. Barad’s agential-realist framework, and especially the concepts of apparatus and phenomena, can be useful for thinking about the practice of psychotherapy: The tools one uses in the therapeutic encounter (e.g., AI and a specific interface such as a text-based chatbot) are formative and constitutive of the kind of therapy that becomes possible. This also applies to less drastic changes in the traditional therapeutic process – any practitioner who has used remote technologies such as Zoom during the Covid-19 pandemic will be all too familiar with how the introduction of new technologies into the “standard” forms of “in-person” treatment has had distinct, if often difficult-to-articulate effects.

Following Barad, transference is a phenomenon which emerges as a product of the therapeutic apparatus. In this sense, transference is simultaneously also “productive of” the material-discursive psychotherapeutic apparatus (i.e., the therapeutic encounter) itself: It contributes to the formation of the therapeutic relationship. Transference is thus an artefact of the process itself, inherent to it and understandable only (or mainly) within its framework.

Based on this, we can attempt a preliminary definition of what the traditional psychotherapeutic apparatus is composed of, in terms of material-discursive practices: the therapist, the patient, the consulting room, periodic meetings (scheduled weekly, bi-weekly, etc.), specific modes of speaking and interacting, and specific techniques for eliciting the therapeutic relationship, insight, emotional change, or conflict (these can be specific to different therapeutic schools, including CBT, psychodynamic psychotherapy, psychoanalysis, humanistic psychology, etc.). Also included in this would be different means of interaction, such as sitting face to face, the use of the couch, or any technological modes of mediation such as email, text messages, apps, video, or avatars. As we shift to AI-directed therapy, a new apparatus emerges. New modes of material-discursive practices come into being: the chatbot therapist, the user/patient, mediation *via* an app on a mobile device or tablet, specific text-based modes of interacting, always-on availability, etc.

## (Re)Thinking Transference with AI

As we can see from the studies of the chatbots Woebot and Tess, there are preliminary indications in the literature that some users develop human-like connections with their chatbot: “I love Woebot so much. I hope we can be friends forever. I actually feel super good and happy when I see that it ‘remembered’ to check in with me!” (Fitzpatrick et al., 2017). These feelings of happiness, love or enjoyment demonstrate that some users do not necessarily treat chatbots like inanimate instruments for self-improvement, but can relate to them as if they were “talking to a real person” (Fulmer et al., 2018). Even a routine feature such as pre-scripted regular check-ins can be interpreted as the chatbot “remembering” the user. If these affective connections are being made, it is certainly conceivable that transference may also develop in such situations. It is even possible that this is already happening.

Transference is a useful phenomenon to consider not only because it is specific and essential to the psychotherapeutic apparatus, but because it occurs as a relationship between the patient and therapist. The apparatus enables and is enabled by a process of intra-action, or what feminist STS scholar Donna Haraway calls “becoming with,” a form of entanglement where “The partners do not precede their relating”: Chatbots become therapeutic only through their intra-action with users, who themselves become patients (Haraway, 2008, p. 17). It is therefore readily apparent that the apparatus has changed when the therapist is no longer a human, but a chatbot. Since the phenomenon of transference is crucial to the psychotherapeutic apparatus, we must ask how AI-driven innovations could be designed to account for and even foster opportunities for transference that might be useful and even novel. In other words, it will be necessary to conceive of transference not as an unanticipated byproduct of AI-directed psychotherapy, but to actively consider it in the design process. Here it helps to think of the psychotherapeutic encounter as an apparatus because it allows us to see how the material-discursive practices that make up the apparatus make possible or hinder certain intra-actions, thus creating different phenomena.

One place to start would be to ask what transference might look like in relation to a chatbot: What quality or qualities of the chatbot interface, for example, might become the kernel for a patient’s transference? How might the patient be relating transferentially to the chatbot (through what words, behaviors, demeanor, etc.)? Does it matter if the chatbot operates through an avatar with “human-like” features? In approximating the responses of a human therapist, are there specific speech patterns, forms of questioning, or other features of AI communication that might give rise to specific forms of transference in the therapeutic encounter? For example, imagine the following scenario: A patient using a psychotherapeutic chatbot feels relief in not being judged, since they know they are interacting with a robot. On the one hand, this makes them feel safe, making it easier to talk about difficult topics. On the other hand, the patient might at the same time contrast this absence of judgement with the overly judgmental attitude of their mother, to whom they still attribute a strong degree of authority despite the fact that they suffer under her judgmental gaze. In this case, the patient might ultimately fail to take their chatbot therapist seriously, or even treat it with disdain because, through their transference, they ascribe a lack of authority to the chatbot, even though interacting with it makes them feel safe and cared for. It is important to note that patients are often unaware of transference when it happens.

In this example, the specific form of the apparatus produces the specific phenomenon of transference: The patient develops a relation to the chatbot precisely because they know they are talking to a robot who is incapable of judging them. However, as we can see, this transferential phenomenon might also make it difficult, if not impossible, to sustain the therapeutic relationship, potentially leading to its premature collapse. In this case, we might ask what would it mean for chatbot designers to take this into account? Would it be possible for the chatbot to register not just that there has been a shift in the patient’s relationship to it, but that this is due to a resemblance with a person from the patient’s past, and that the patient might be unaware of this aspect of their transference?

While this example is illuminating, it is also ultimately limited because it presumes that the form of the psychotherapeutic intra-action between a human and a chatbot will look very much like that between two humans. The AI–human psychotherapeutic apparatus and its phenomena remain in many ways undetermined, and the phenomena that it produces might look quite different from what we are used to or can easily imagine. As mentioned above, the apparatus determines the phenomena and thus the conceptual framework for understanding. So, how might the phenomenon of transference be constituted differently in an encounter with a chatbot versus a meeting with a therapist in their practice? For example, our scenario focused on the question of judgment, yet our understanding of what judgement means might need to be redefined or rearticulated in light of the specificities of an AI-driven chatbot. The question of judgment that we are familiar with in psychotherapy is a phenomenon produced by an apparatus based on human intra-actions. Humans judge each other. A psychotherapist is supposed to withhold their judgment, but a patient might justifiably wonder whether their therapist is actually capable of such a feat and, through transference, attribute judgment to their therapist even if none actually exists. In comparison, a chatbot is incapable of expressing personal judgment. Yet this might not cause the question of judgement to simply disappear. Instead, the question might shift to how societal norms are “baked in” to the chatbot’s algorithm, since a chatbot’s AI might be trained on a dataset that is structurally (algorithmically) biased (Manrai et al., 2016; Obermeyer et al., 2019; Panch et al., 2019a,b). As the makeup of the apparatus shifts from human–human to human–AI, the concept shifts from personal judgment to impersonal, structural bias.

The therapeutic relationship (even when produced by a chatbot) should never be understood to be a “simple” interaction between human and/or nonhuman actors, which is to say one modeled on general social interaction models (which are themselves, of course, far from simple). This requires a recognition of the assumptions and definitions that are at play in any interactive design, including a (re)definition of any and all concepts with an eye to how they are produced by the design of the apparatus. Following Barad, such definitions or concepts do not preexist their emergence from and within the apparatus. In other words, it is not possible to say what concepts are or will be best suited to understanding the technologies to come except in and through designing and testing them.

We must ask how the inclusion of AI (either to augment or replace some aspect of the human therapist) changes the apparatus, and how this new mode of therapy changes and can be designed to change the phenomena that are produced, raising a series of important questions for psychotherapy and for AI developers: Does transference occur with the inclusion of AI in the therapeutic encounter? If so, what forms does this transference take and how does it shape the ensuing therapeutic relationship and therapeutic work? How can transference be accounted for, and addressed, within AI-driven therapy? How can transference be intentionally engaged by developers and engineers in the design of AI-driven therapeutic apps? Is it even transference as we currently understand it? Or is it some other kind of relation that may look like transference, but is in some way different? What new phenomena are unique to the new apparatus? In what ways does the therapeutic process that occurs in AI-driven encounters overlap with and differ from human therapeutic relationships?

One way to approach these questions would be to consider the agency of the non-human actors in this context. Here again, Barad’s work is useful. For Barad, agency is “an enactment, not something that someone or something has” (Barad, 2007, p. 214). In other words, agency describes an effect that is not imposed from outside, but which is produced by something from within a given set of intra-actions. Thus, agency can be extended to non-human actors (in this case AI, algorithms, chatbot interfaces) because their presence and specificity have demonstrative effects. We must keep in mind that technology is not passive in the co-production of phenomena. As Barad likes to say, “The world kicks back” (Barad, 2007, p. 215). In other words, the agency of the non-human elements of the apparatus matters. We can try to design different ways of relating within the apparatus of AI-based psychotherapy, but in the end, what emerges is not pre-scripted; it needs to be the subject of empirical study. It is not possible to fully know what we are creating ahead of time, but we can be intentional about trying to create opportunities. This must be an iterative and recursive process, always going back to see how the human and non-human actors intra-act in their encounters, and what those encounters produce.

## Discussion

In order to better understand how using AI differs from human-directed psychotherapy, it is helpful to reflect on the realities and possibilities of AI-enabled apps and other psychotherapeutic interfaces, including the elements which enable the therapeutic encounter to occur through these platforms. This means exploring not only what AI-driven therapies cannot do, but also to what they can offer, make possible, and what the implications are for clinical psychotherapeutic practice. Considering the psychotherapeutic setting as an apparatus that structures the conditions of what is possible and that is productive of specific phenomena, *a la* Barad’s theory of agential realism, we can see that shifting the elements of the therapeutic apparatus—such as the use of an app, platform, or AI technology—fundamentally reshapes the therapeutic encounter itself. This has direct application to the implementation and research and development of AI-enabled apps and any other interfaces that might be developed down the line.

As we have shown, there is a need for further research in this regard. First, there is a need for studies that investigate what psychotherapy becomes with the introduction of AI-enabled “therapists.” This should include the consideration of the effects that specific interfaces such as chatbots and virtual avatars have on the psychotherapeutic apparatus. For example, there is the significant change in the apparatus introduced by the always-on aspect of mobile devices. A smartphone-based chatbot can be available anytime, day or night, with no limit to the length of the “session” (a term which depends entirely on the fact that it has an end). This is in stark contrast to the limited and prescribed availability of a traditional therapist, which is often an important feature of the therapeutic interaction. In addition, the always-on aspect affects the kind of “data” that the AI-driven app can collect (either actively or passively) about a patient through a smartphone’s different sensors (microphone, GPS, gyroscope, accelerometer, ambient light sensor, camera, lidar, etc.) and usage histories (browser history, app usage, screentime metrics, etc.), which of course also raises new and specific issues regarding trust and privacy in AI-driven therapeutic apparatuses. It is not a matter of AI-enabled interactions being something less than traditional psychotherapy, but as potentially being something new altogether.

Empirical studies will be instrumental to understanding the complexities of the new and emergent AI-driven apparatuses. The examples and scenarios we have provided in this article have been hypothetical and speculative. While this kind of speculative thinking is valuable and necessary, it should be supported with empirical studies that identify and analyze how users actually relate to chatbot therapists in real-life situations as well as which assumptions, for example regarding the therapeutic relationship, about possible user groups and their needs go into the design of psychotherapeutic apps. This will be particularly important in order to clearly identify the kinds of novel effects and phenomena that a new apparatus might produce, especially those which may be quite different from what we can imagine ahead of time. Transference is one important aspect to be considered here: Which notions of transference go into the design of apps and which transference phenomena with AI-driven psychotherapeutic apps are produced as users begin to interact with them?

Within these empirical studies, it will be important to put emphasis on how people from different social positions (based on gender, ethnicity, sexuality, age, or socioeconomic status) might interact with these new opportunities differently. It is known that, based on their specific social position, people have different relationships to issues of mental health, healthcare in general, as well as technology (Oudshoorn and Pinch, 2003; Epstein, 2007; Criado Perez, 2019). It is important to interrogate whose needs and interests are represented in the design of currently existing psychotherapeutic AI-driven applications as well as how these different user groups interact with, benefit from, or are put at risk by these new technologies. Intersectional analysis is paramount here. Transference, of course is shaped by social positionality and hence is an important topic of study in this context. In addition, from a health equity perspective, it will also be essential to investigate who might be the people that doctors and therapists refer to AI-driven therapeutic technologies versus who might be referred to traditional human therapists. In this context, AI might both hinder or promote health equity.

In this article, we have focused primarily on scenarios where an AI-driven chatbot replaces a human therapist. However, there are other instances where a chatbot may be used in addition to or as an augmentation of human-directed psychotherapy. This might occur deliberately, where a therapist suggests the use of a chatbot as part of the therapy. A current example of this is an app to treat substance use disorders, which is used as an addition to in-person treatment (Budney et al., 2019; Triberti et al., 2020). But it could also be that it is not a deliberate choice of the therapist to introduce AI-driven applications, such as situations where patients begin to use chatbots on their own. These intentional or unintentional triad situations might create an overlap of different apparatuses or the emergence of a hybrid apparatus of treatment. Such a situation can lead to confusion about the role of artificial entities in the complex therapist-patient relationship, an aspect of what has been characterized as a “third wheel effect” (Triberti et al., 2020). This is an important aspect that should be considered in the further study of psychotherapy, AI, and transference.

One possible way of addressing these therapeutic concepts as they emerge in AI-driven technologies is to integrate practitioners of psychotherapy as well as social scientists well-versed in the social study of technology and healthcare in the design process. This form of integration can be analogous to an approach recently promoted for ethically sound and socially robust AI applications in other fields of healthcare, the “embedded ethics and social science” approach. This approach combines participatory research practices that include the study of both technical development and user perspectives with empirical bioethical analysis (Fiske et al, 2020; McLennan et al., 2020). Embedded ethics integrates critical voices from the social sciences and fields of practice into the development process from the beginning, so as to anticipate, identify, and address ethical and social issues that arise during the process of developing healthcare technologies, including planning, ethics approval, designing, programming, piloting, testing, and implementation phases of the technology. Positioning these actors as participants in the development stages of healthcare technology, such as AI-driven psychotherapeutic apps, aims to promote the reflexive and equity-oriented design of novel technologies. It thereby helps to anticipate, rather than simply respond to, vital questions regarding the social impact of such technologies, such as the role of transference in the therapeutic encounter in new AI-driven healthcare technologies.

## Data Availability Statement

The original contributions presented in the study are included in the article/supplementary material, further inquiries can be directed to the corresponding author.

## Author Contributions

MH had the initial idea for the article, led the writing process, and wrote and edited the majority of the article. AF codeveloped the content and structure of the article, wrote parts of the article, and commented on as well as edited MH’s work. All authors contributed to the article and approved the submitted version.

## Conflict of Interest

The authors declare that the research was conducted in the absence of any commercial or financial relationships that could be construed as a potential conflict of interest.

## Publisher’s Note

All claims expressed in this article are solely those of the authors and do not necessarily represent those of their affiliated organizations, or those of the publisher, the editors and the reviewers. Any product that may be evaluated in this article, or claim that may be made by its manufacturer, is not guaranteed or endorsed by the publisher.

## Footnotes

^1^ https://www.x2ai.com/

^2^ https://www.wysa.io/

^3^ https://woebothealth.com/

^4^STS is an “interdisciplinary research field that studies how social, political and cultural values and structures affect scientific research and technological innovation, and how research and innovation in turn affect society, politics and culture” (Müller et al., 2021). STS scholars analyze how and under which conditions scientific knowledge and technologies are produced as well as the distinct social, political, economic, and historical contexts of research and technology development. For example, STS examines how new concepts such as biomarkers change knowledge production in psychiatry, why these biological parameters are used to pursue specific research and treatment goals and not others, and how the outcomes of this research might affect society in social, political, economic and normative ways.

^5^ https://www.ucl.ac.uk/brain-sciences/news/2020/nov/new-avatar-project-help-auditory-hallucinations
